# Beyond structure prediction: Translating D‐I‐TASSER models into functional and mechanistic hypotheses

**DOI:** 10.1002/ctm2.70879

**Published:** 2026-10-14

**Authors:** Yujie Mo, Tianrui Jia, Yang Zhang

**Affiliations:** ^1^ Department of Computer Science, School of Computing National University of Singapore Singapore Singapore; ^2^ Department of Biochemistry, Yong Loo Lin School of Medicine National University of Singapore Singapore Singapore; ^3^ Cancer Science Institute of Singapore National University of Singapore Singapore Singapore; ^4^ Center for AI and Computational Biology, Suzhou Institute of Systems Medicine Chinese Academy of Medical Sciences & Peking Union Medical College Suzhou China

1

Deep learning has revolutionised protein structure prediction, with AlphaFold demonstrating near‐experimental accuracy for many targets and enabling structure prediction at proteome scale.^1^, ^2^ This advance has expanded the role of predicted structures in functional annotation, mechanistic studies, and structure‐guided experimental design. However, several significant challenges remain to be addressed. First, since many current AI models are trained on evolutionary profiles, proteins lacking detectable homologous sequences remain difficult to model.^3^ In addition, for multidomain targets, accurate models of individual domains do not necessarily yield a correct full‐length structure, because errors in their relative orientation can obscure interfaces and other functional relationships. Such errors are not merely geometric: they can change the inferred spatial relationships among ligand‐binding regions, protein–protein interfaces and regulatory sites, and therefore alter the biological interpretation derived from a predicted structure. This distinction is particularly important in translational research, where structural models are increasingly used to formulate mechanistic hypotheses and guide subsequent experiments.

To address these challenges, we developed D‐I‐TASSER,^4^ a hybrid framework that combines deep learning with physics‐inspired conformational sampling for atomic‐level protein structure prediction. Rather than accepting the output of a single neural network as the final structural prediction, D‐I‐TASSER uses replica‐exchange Monte Carlo simulations to assemble structural models under a composite force field that integrates multiple complementary deep‐learning‐derived spatial restraints, threading‐derived template information, and knowledge‐ and physics‐based energy terms.^5^ The resulting conformational ensemble is clustered to identify dominant structural states, which are expected to correspond to low‐free‐energy regions of the conformational landscape.^6^ To enhance the collection of evolutionary information, D‐I‐TASSER utilises DeepMSA2 to construct deep multiple‐sequence alignments (MSAs) through iterative searches of genomic and metagenomic databases, thereby improving both deep‐learning‐based restraint prediction and remote template identification.^7^ For multidomain proteins, D‐I‐TASSER explicitly partitions the sequence into domains, generates domain‐level MSAs, templates, and spatial restraints, and then recombines them to guide full‐chain assembly. After model clustering and refinement, the resulting structures can be further analysed for structure‐based protein function prediction, as summarised in Figure 1. This hybrid strategy therefore represents not simply another end‐to‐end neural network, but a complementary approach in which deep learning guides an explicit conformational search.

**FIGURE 1:**
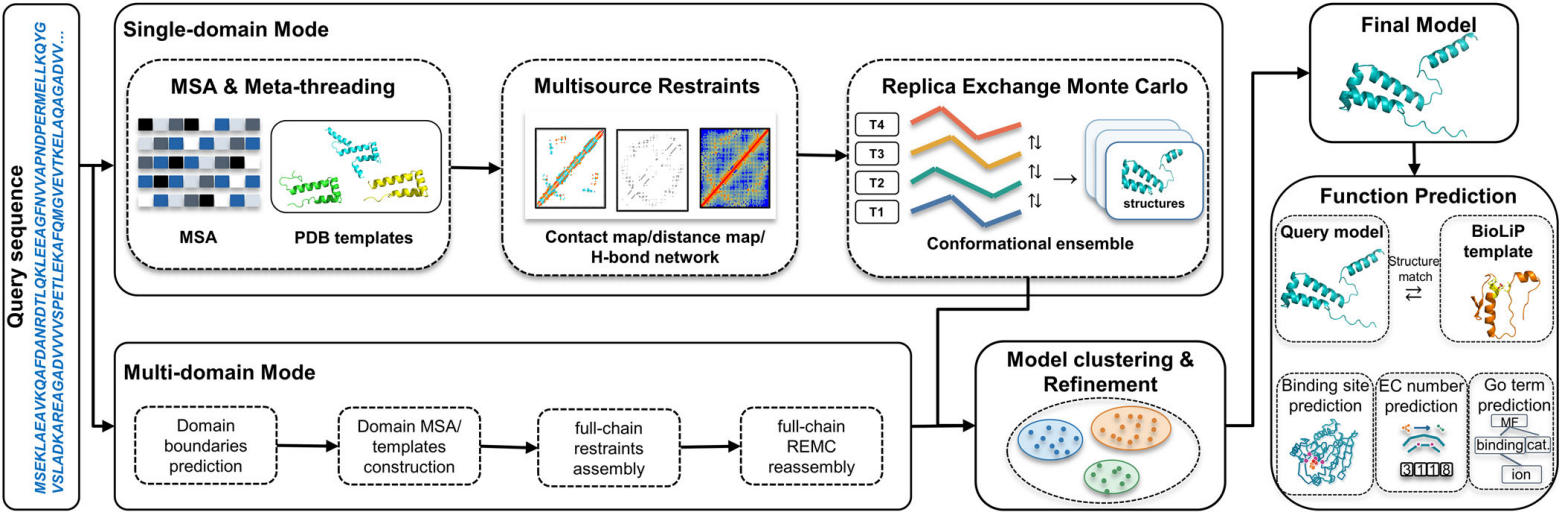
Overview of the D‐I‐TASSER workflow for protein structure and function prediction. Given a query sequence, D‐I‐TASSER constructs deep MSAs, identifies templates by meta‐threading, and then integrates deep‐learning‐derived spatial restraints with a knowledge‐ and physics‐based force field into replica‐exchange Monte Carlo simulations, with explicit domain partitioning and full‐chain reassembly for multidomain proteins. After that, D‐I‐TASSER performs clustering and refinement to obtain the final models, which are then analysed with COFACTOR for function prediction.

Benchmark studies and the results of the 15th community‐wide Critical Assessment of Protein Structure Prediction (CASP15)^8^ demonstrated D‐I‐TASSER's leading performance. In the benchmark testing, across 500 hard domains lacking significant homologous templates, D‐I‐TASSER achieved a 5% higher average template modelling (TM) score than AlphaFold2, with the clearest gains among the most difficult targets. Ablation analyses indicated that this improvement reflected the combined contributions of deeper multiple‐sequence alignments, complementary deep‐learning‐derived restraints, and conformational assembly. Across 230 multidomain proteins, D‐I‐TASSER improved the full‐chain TM‐score by 12.9% relative to AlphaFold2. This improvement was primarily attributed to the domain‐partitioning and reassembly procedure, which improved interdomain organisation rather than simply refolding individual domains. The blind CASP15 assessment independently supported these findings, with D‐I‐TASSER ranking first among server groups in both regular modelling and interdomain modelling. These gains are particularly relevant for challenging domains and multidomain proteins, where AI‐predicted models with incorrect interdomain organisation may lead to misleading mechanistic hypotheses.

Beyond its benchmark and CASP15 performance, the human‐proteome analysis demonstrated the scalability and practical reach of D‐I‐TASSER. Specifically, D‐I‐TASSER was applied to 19 512 human proteins, and approximately 81% of domain‐level models and 73% of full‐chain models were estimated to have correct global folds.^4^ Importantly, the resulting model set was highly complementary to the AlphaFold2 human‐proteome models, with a substantial subset of proteins judged foldable by only one of the two approaches. The significance of this complementarity lies not simply in increasing structural coverage, but in extending proteome‐wide sequence‐to‐structure‐to‐function analysis to proteins that remain difficult for the current modelling paradigm. From a translational perspective, this complementarity may be more important than viewing D‐I‐TASSER simply as a competitor to AlphaFold. When one modelling paradigm is uncertain, an independently generated structural hypothesis can help identify predictions that are robust across methods and cases in which alternative structural interpretations warrant further experimental investigation.

We then examined whether these predicted structures could support testable hypotheses about protein function. Specifically, the predicted models were analysed with COFACTOR^9^ to infer Gene Ontology terms, Enzyme Commission classifications, and ligand‐binding sites across the human proteome. As a retrospective proof of principle, acetyl‐CoA acetyltransferase illustrates how an accurate structural model can recover functionally relevant molecular features (Figure 2a and b): the predicted CoA pose had a root‐mean‐square deviation (RMSD) of 0.74 Å relative to the experimental pose, and 22 of the 23 observed ligand‐binding residues were recovered.^4^ Importantly, these functional predictions were derived from the D‐I‐TASSER model, with the experimental structure used only for retrospective validation, supporting the application of this approach to proteins for which experimental structural information is unavailable or incomplete. Predicted structural features can guide functional investigation by identifying ligand‐binding pockets for biochemical or mutagenesis studies, protein or interdomain interfaces for interaction assays, and the structural context of disease‐associated variants relative to functional sites. For multidomain proteins, accurate interdomain organisation can further reveal functional relationships between residues that are distant in sequence but brought together in three‐dimensional space. Such predictions do not constitute clinical evidence or replace experimental validation; rather, they can narrow the experimental search space and translate structural predictions into mechanistically informed, testable hypotheses relevant to disease mechanisms and therapeutic investigation.

**FIGURE 2:**
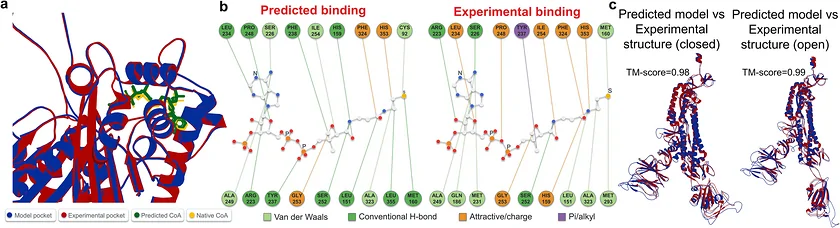
Functional and conformational insights from D‐I‐TASSER models. (a) A case study of CoA binding to acetyl‐CoA acetyltransferase (UniProt ID: Q9BWD1). The D‐I‐TASSER model, experimental structure, COFACTOR‐predicted CoA pose, and native CoA pose are shown in blue, red, green, and yellow, respectively. (b) Comparison of the COFACTOR‐predicted (left) and experimental (right) CoA‐binding pockets, highlighting representative residue‐CoA interactions. Interaction lines indicate different contact types. (c) Superposition of the D‐I‐TASSER open‐state and closed‐state models of the SARS‐CoV‐2 spike protein with the corresponding experimental structures (PDB ID 6VYB and 6VXX).

One important limitation of many current end‐to‐end deep‐learning approaches is that they typically produce a single static structural model, whereas proteins in living cells often populate multiple conformational states in response to ligand binding, molecular interactions, and the cellular environment. Figure 2c illustrates the potential of D‐I‐TASSER to move beyond such a static representation. By integrating deep‐learning‐derived spatial restraints with energy‐based conformational sampling, D‐I‐TASSER explicitly explores conformational space under a hybrid energy function and generated models corresponding to both the open and closed states of the SARS‐CoV‐2 spike protein.^4^ This capability is important because protein function often depends on transitions among functional states rather than on a single static structure. For functional annotation, conformational ensembles may reveal state‐dependent binding sites, interfaces, and regulatory regions that are missed in a single structure. For drug design, they may expose transient or allosteric pockets and provide alternative receptor states for structure‐based screening. More broadly, extending conformational sampling toward accurate modelling of protein folding and dynamics‐including the pathways and energetics connecting different states‐represents an important frontier for linking protein structure to molecular mechanism and function.^10^

Despite these advances, important challenges remain before predicted structures can be routinely used in functional and translational studies. D‐I‐TASSER still depends substantially on the availability and quality of evolutionary information, making orphan proteins and other shallow‐MSA targets particularly challenging.^4^ Accurate interdomain organisation also remains more difficult than individual‐domain modelling, while intrinsically disordered regions and protein complexes pose additional challenges. Equally important is reliable estimation of model confidence and uncertainty, since even structurally plausible models may lead to incorrect functional interpretations. Predicted structures should therefore be viewed as testable molecular hypotheses whose value depends not only on structural accuracy, but also on an understanding of their uncertainties and subsequent experimental validation.

Future work should broaden the applicability and reliability of D‐I‐TASSER through improved evolutionary information, more accurate modelling of multidomain proteins and molecular complexes, and systematic integration of complementary structures and spatial restraints from different AI models. A more fundamental frontier, however, is to move beyond static structure prediction toward understanding protein folding and conformational dynamics‐the pathways, energetics, and populations through which proteins reach and transition among functional states.^10^ Such dynamics are central to molecular function, as proteins often operate through transitions among conformational states rather than through a single static structure. Integrating dynamic modelling with structure‐based functional annotation may therefore provide a more complete link from sequence to structure, dynamics, and function, revealing state‐dependent functional features that cannot be captured by a single static model and opening new opportunities for understanding disease mechanisms and structure‐based drug design. Ultimately, progress should be judged not only by improvements in benchmark accuracy, but also by whether computational models reveal molecular mechanisms and generate experimentally testable hypotheses, thereby shortening the path from protein sequence to functional understanding, experimental validation, and, where appropriate, translational application.

## AUTHOR CONTRIBUTIONS

Y.Z. conceived the project, supervised the work, and critically revised the manuscript. Y.M. drafted the manuscript. T.J. contributed to figure preparation and manuscript revision. All authors read and approved the final manuscript.

## CONFLICT OF INTEREST STATEMENT

The authors declare no conflict of interest.

## ETHICS STATEMENT

Not applicable. This article does not report new studies involving human participants or animals.
